# Polyphenols: Multipotent Therapeutic Agents in Neurodegenerative Diseases

**DOI:** 10.1155/2013/891748

**Published:** 2013-06-06

**Authors:** Khushwant S. Bhullar, H. P. Vasantha Rupasinghe

**Affiliations:** Department of Environmental Sciences, Faculty of Agriculture, Dalhousie University, Truro, NS, Canada B2N 5E3

## Abstract

Aging leads to numerous transitions in brain physiology including synaptic dysfunction and disturbances in cognition and memory. With a few clinically relevant drugs, a substantial portion of aging population at risk for age-related neurodegenerative disorders require nutritional intervention. Dietary intake of polyphenols is known to attenuate oxidative stress and reduce the risk for related neurodegenerative diseases such as Alzheimer's disease (AD), stroke, multiple sclerosis (MS), Parkinson's disease (PD), and Huntington's disease (HD). Polyphenols exhibit strong potential to address the etiology of neurological disorders as they attenuate their complex physiology by modulating several therapeutic targets at once. Firstly, we review the advances in the therapeutic role of polyphenols in cell and animal models of AD, PD, MS, and HD and activation of drug targets for controlling pathological manifestations. Secondly, we present principle pathways in which polyphenol intake translates into therapeutic outcomes. In particular, signaling pathways like PPAR, Nrf2, STAT, HIF, and MAPK along with modulation of immune response by polyphenols are discussed. Although current polyphenol researches have limited impact on clinical practice, they have strong evidence and testable hypothesis to contribute clinical advances and drug discovery towards age-related neurological disorders.

## 1. Introduction

Neurodegenerative disorders such as Alzheimer's disease (AD), stroke, and Parkinson's disease (PD) represent a major clinical problem in the developed countries [1, 2] and are major economic burdens for health care systems [3]. Dietary [4], genetic, and molecular factors [5] are important determinants in progression and intervention of neurodegenerative diseases. AD is a common cause of dementia and mortality in the United States. Total numbers of reported deaths due to AD have increased in past years, and it is among 10 leading causes of deaths in the United States [6]. Amyloid-*β* (A*β*) peptides derived from amyloid precursor protein (APP) via *γ*-secretase and *β*-secretase cleavage are hallmarks of AD [7]. Cellular prion protein (PrP(C)) [8] and oxidative stress [9] mediate A*β* neurotoxicity, and the latter contributes to neuronal death by lowering intracellular glutathione. Along with A*β*, tau protein alteration in neuronal microtubules also contributes to the pathology of AD [10]. Abnormal phosphorylation and aggregation of tau protein leads to neural dysfunction and leads to pathological events which cause neuronal dysfunction in AD [11]. Failed clearance of A*β* aggregates resulting from impaired autophagy may also contribute to AD [12]. AD is also characterized by elevated peripheral blood cytokine concentrations for interleukin- (IL-) 6, tumor necrosis factor alpha (TNF-*α*), IL-1*β*, transforming growth factor beta (TGF-*β*), IL-12, and IL-18 suggestive of a pro-inflammatory response in AD pathology [13]. 

Multiple sclerosis (MS) is another neurodegenerative disease characterized by chronic inflammation accompanied by demyelination of neurons in brain [14]. MS is characterized by symptoms like mood disorder, fatigue, vision changes, muscle weakness, and motor changes [15]. Chemokines like IL-17, chemokine (C-C motif) ligand 17 (CCL17), and CCL20 are suggested as major mediators in MS neuroinflammation and pathology [16]. 

Stroke is the third leading cause of mortality, loss of cognitive functions, and heavy socioeconomic burden in the United States [17]. Similar to MS, stroke or cerebral ischemia is a pathological condition accompanied by inflammation and immune system disease [18]. One minute of cerebral ischemia is estimated to destroy approximately 2 million neurons and 14 million synapses [19]. Like AD and MS, inflammatory cytokines including TNF-*α* and IL-1 and IL-6 play modulatory role in stroke pathology [20]. Transcription factor, nuclear factor kappa B (NF*κ*B), is an important regulator in pathology of inflammation and neuronal cell survival, as its activation leads to cell death in cerebral ischemia [21]. 

PD is a progressive neurodegenerative disease; its familial forms are characterized by mutations of six genes including clinically important ATP13A3 resulting in cognitive impairment and depression [22]. Some PD cases also involve changes in micro-RNA and *α*-synuclein level in patients [23]. Like other neurodegenerative diseases, PD also involves elevated levels of proinflammatory cytokines monocyte chemoattractant protein-1 (MCP-1), CCL-5, macrophage inflammatory protein-1*α* (MIP-1*α*), IL-8, interferon-gamma (IFN*γ*), IL-1*β*, and TNF*α* [24]. After examining cytokines in 52 PD patients, researchers [25] suggested involvement of TNF-*α* in production and maintenance of nonmotor symptoms. Mitochondrial dysfunction also plays an important role in pathogenesis of PD [26] similar to AD [27], MS [28], and stroke (CI) [29].

Huntington's disease (HD) is another neurological disorder causing cognitive impairment, accompanied by oxidative stress and mitochondrial dysfunction. It results from increased number of CAG triplet nucleotide repeats and expanded polyglutamine region of huntingtin protein [30]. HD pathology leads to elevated levels of chemokines like eotaxin-3, MIP-1*β*, eotaxin, MCP-1, and MCP-4 [31]. 

The pathophysiology of neurological disorders is also accompanied by alterations in electrical activity of neurons at cellular level. The voltage gated ion channels are required for action potential generation and its propagation in neurons, and their dysfunction contributes to pathology of neurodegenerative diseases. The brain electrical activity is significantly changed in AD and dementia leading to impaired verbal memory and cognitive skills [32]. The Kv3 subfamily of K^+^ channel subunits, which possess ability of fast repolarization of action potential [33], are compromised and slowed down in AD [34]. The upregulation of the K(v)1.3 potassium channel also plays important role in immunopathogenesis of multiple sclerosis and presents therapeutic option by blocking Kv channels [35]. As sodium channels (Na_v_1.8, Na_v_1.5) play an important role in electrical activity of neurons, their overloading is thus an important mediator in axonal degeneration in MS [36, 37]. Such electric disturbances are also found in PD [38] which impose energy burden by Ca^2+^ entry through L type voltage-dependent channels [39]. Similarly, sodium and potassium channel abnormalities are proposed to contribute to HD pathogenesis [40, 41].

**Figure 1:**
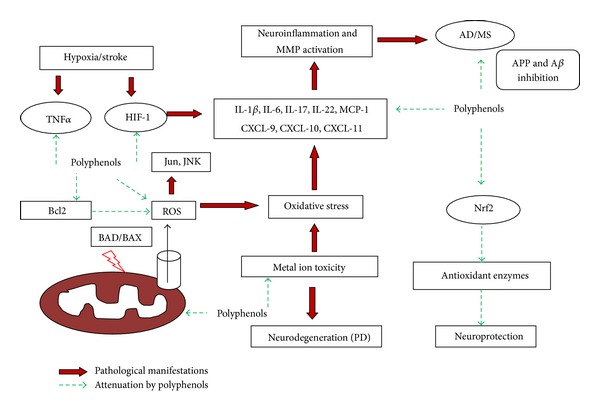
Neuroprotection by polyphenols against neurological disorders.

There are a few clinically relevant medicines and therapies available for AD, HD, MS, PD, and stroke. A few clinically active, yet expensive, options such as acetylcholinesterase inhibitors, interferon *β*-1a, levodopa, tetrabenazine, and tissue type plasminogen activator (tPA) are available for AD [42], MS [43], PD [44], HD [45], and stroke [46], respectively. In wake of these pathologies and limited clinical treatments, alternative and preventive therapeutics are required which can control the occurrence and progression of neurodegenerative diseases. All the neurodegenerative diseases discussed above have the common features of pathogenesis which include cytokine changes, genetic alterations, immunomodulation, inflammation, mitochondrial dysfunction, oxidative stress, prions, and protein dysfunction. Recent research has shown that dietary polyphenols target the pathological manifestations of neurological disorders with their ability to cross blood-brain barrier [47] as they control neuronal disease pathogenesis at a molecular and symptomatic level by targeting these common features of neurodegeneration pathology. Polyphenols are naturally occurring phytochemicals found in fruits and vegetables, exhibiting strong neuroprotective properties [48]. Important dietary sources of polyphenols include apples, berries, cocoa, herbs, red wines, seeds, onions, and tea [49]. Dietary polyphenols have also been implicated in prevention of oxidative damage and LDL oxidation [50–52]. This review briefly outlines the pharmacological role of polyphenols in preventing neurodegenerative diseases based on the most recent scientific literature (Figure 1).

## 2. Polyphenols and Pharmacological Properties

### 2.1. Alzheimer's Disease and Dementia

Polyphenols exhibit neuroprotective properties including therapeutic action in AD and dementia. Green and white tea extracts have been shown to inhibit acetylcholinesterase which indicates their potential in treatment of age-related disorders such as AD [53]. Green tea polyphenols protect primary rat cortical neurons against A*β*-induced cytotoxicity [54]. In mouse model studies [55], polyphenols of grapes improved cognitive functions in mouse model of AD. As well, epicatechin metabolite 3′-*O*-methyl-epicatechin-5-*O*-*β*-glucuronide had improved synaptic transmission through cyclic adenosine monophosphate (cAMP) response element binding protein. In transgenic mice model studies, grape seed polymeric polyphenol extract has been shown to inhibit oligomerization of A*β* peptides and contributed to reduction in cognitive impairments in transgenic mice [56]. A similar study showed that polyphenols of grapes exhibited potential in neutralizing abnormal folding of tau proteins [57]. Earlier studies using animal models [58, 59] also confirm anti-A*β* action of grape seed polyphenols. 

Resveratrol, a polyphenol abundant in grapes and red wines, inhibited A*β* 42 fibril formation [60] and protected from A*β* neurotoxicity by inhibiting inducible nitric oxide synthase inhibition [61]. Resveratrol, with possibly high bioavailability in lipid core nanocapsules, exhibited therapeutic action in AD [62]. Flavonoid fisetin and its analogues also inhibited A*β* fibril formation and have emerged as new drug candidates for AD treatment [63]. Morin (2′,3,4′,5,7-pentahydroxyflavone) has shown to prevent neuronal cell death by protecting neurons against tau hyperphosphorylation induced by A*β* [64]. Similarly, in transgenic mouse model studies [65], tannic acid has displayed the attenuation of A*β* deposition by decreasing cleavage of *β*-carboxyl terminal amyloid precursor protein (APP) fragment and controlled neural inflammation. A flavonoid, 7,8-dihydroxyflavone, has been shown to improve cognitive abilities in 5XFAD transgenic mouse model of AD by activation of tyrosine receptor kinase B leading to reduction in *β*-secretase enzyme levels and amyloid beta (A*β*) synthesis [66]. Similarly, liquiritigenin improved memory in Tg2576 mice model of AD, as it attenuated astrocytosis and decreased the Notch-2 expression as the latter can contribute to neuronal decay [67]. Unlike resveratrol, quercetin and rutin not only inhibited A*β* formation but also disaggregated A*β* fibrils in AD studies [68]. Both compounds also prevented scopolamine-induced amnesia in animal model systems [69]; however, resveratrol did not reverse scopolamine-induced deficit [70]. Rutin has been found to control oxidative stress, malondialdehyde, and glutathione disulfide formation in SH-SY5Y neuroblastoma cells. Rutin has also attenuated the inflammatory cascade by decreasing cytokines like TNF-*α* and IL-1*β* [71]. Ferulic acid, a phenolic acid, has also exhibited higher neuroprotection against A*β* toxicity than quercetin [72]. Recent research findings have shown that polyphenols have therapeutic relevance in both cell and animal model studies. The ability of polyphenols to improve synaptic transmission by elevating cAMP, target multiple signaling pathways, and reduce A*β* toxicity suggests their therapeutic utility for age-related disorders like AD and dementia.

### 2.2. Multiple Sclerosis

Multiple sclerosis is a neurodegenerative disease characterized by autoimmune-mediated demyelination in the CNS resulting in paralysis and cognitive deficits. MS therapies can reduce inflammation and downregulate immune function [73]. Resveratrol, a silent mating type information regulation 2 homolog1 (SIRT1) activator, has exhibited prevention of neural loss without immunosuppression in experimental autoimmune encephalomyelitis (EAE) model of MS [74]. Pharmaceutical grade formulation of resveratrol SRT501 was found to attenuate neural damage in EAE through SIRT1 activation [75]. Cell culture studies [76] have also shown SIRT1-mediated neuroprotection by resveratrol. Quercetin was found to control immune response via modulation of IL-1*β* and TNF-*α* and reduced the proliferation of peripheral blood mononuclear cells isolated from multiple sclerosis patients [77]. Epigallocatechin-3-gallate (EGCG) exhibited neuroprotective effects by modulating neuroinflammation and attenuating neural damage [78]. Quercetin [79], apple polyphenols [80], myricetin, and piceatannol [81] have also activated SIRT1, thus exhibiting potential in MS treatment. Earlier studies have also shown [82] that flavonoids limit demyelination in MS suggesting their potential against neuro-inflammation and related disorders. Preclinical data has shown that polyphenols exhibit potential to block neural inflammation and damage by activation of SIRT1 pathway along with modulation of inflammatory cytokines. The potential of polyphenols on limiting demyelination makes them prospective therapeutics in age-related MS and amyotrophic lateral sclerosis (ALS).

### 2.3. Ischemic Stroke

Various epidemiological studies suggest that diet rich in polyphenols can extend neuroprotection and lower the risk and severity of stroke, the third leading cause of mortality [83]. Experimental evidence using rodent and cellular models also indicates neuroprotective potential of dietary polyphenols in cerebral ischemia. Green tea polyphenol, EGCG, has exhibited neuroprotective action by downregulation of matrix metalloproteinases (MMP) in mice model of cerebral ischemia [84]. Green tea polyphenols have also been found to protect neurons against hypoxia-induced ischemic injury by controlling inflammation cascade and attenuating decline in transmembrane potential [85]. Quercetin has been found to attenuate ischemic injury by controlling acid-sensing ion channel led calcium dysregulation and lipid peroxidation in neurons [86]. Another study with similar experimentation has supported neuroprotective role of quercetin, based on its ability to block sodium channels [87]. Quercetin with similar antioxidant therapy to green tea polyphenols has reduced the level of MMP-9 and attenuated blood-brain barrier disruption in cerebral ischemia (CI) studies [88]. Researchers have also hypothesized neuroprotective action of quercetin in CI to be based on its inhibitory action against MMP [89]. Rutin has been found to control neural damage in CI through downregulation of p53, a protein which leads to necrosis in stroke [90]. It has also shown the attenuation of glutathione peroxidase, glutathione reductase, and inflammatory cytokines in rodent model of ischemic stroke [91]. In addition, resveratrol has been found to extend protection against ischemic injury by improving brain energy metabolism and controlling oxidative stress during ischemia injury in animal model studies [92], along with the modulation of release of multiple therapeutic neurotransmitters and neuromodulators during ischemic injury [93]. The flavonoid fisetin has shown neuroprotective action during cerebral ischemia as it stopped infiltration of macrophages and dendritic cells into ischemic hemisphere, thus controlling neural inflammation and damage [94]. Another flavonoid baicalin has been shown to reduce ischemic stroke damage by targeting multiple therapeutic targets like MMP-9 [95], caspase-3, oxidative stress [96], and p38 mitogen-activated protein kinase (MAPK) [97] and by downregulating toll-like receptor (TLR2/4) pathway [98]. The experimental data reveals that polyphenols may prevent, attenuate, or slow down, via multiple mechanisms, the course of stroke and age-related neural disorders. Since the risk for stroke increases with age, consumption of polyphenol rich diet seems to be an important preventive strategy. 

### 2.4. Parkinson's Disease (PD)

PD is a neurodegenerative disease accompanied by inflammation and oxidative stress resulting in loss of dopaminergic neurons in the substantia nigra [137]. Polyphenols with their ability to attenuate oxidative stress and inflammation present therapeutic option in neurodegenerative disease. Resveratrol has been shown to inhibit the loss of dopaminergic neurons in rat model of PD [76]. Resveratrol has also been shown to reduce neural inflammation in PD by lowering mRNA levels of cyclooxygenase-2 (COX-2) and TNF-*α* mRNA in the substantia nigra [100] along with attenuation of oxidative stress, lipid peroxidation, and protein carbonyl (PC) in rat model of PD [138]. Oxyresveratrol has demonstrated attenuation of neural damage in SH-SY5Y cells by elevating levels of SIRT1 and downregulating expression of caspase-3, c-Jun N-terminal kinase (JNK), and c-Jun transcription factors [139]. Ferulic acid, like oxyresveratrol, has demonstrated neuroprotective effect via downregulation of JNK pathway [140]. Quercetin administration to neurons attenuated 1-methyl-4-phenylpyridinium (MMP) evoked microglia activation, which is a precursor for PD pathogenesis [124]. Studies have also shown that quercetin promises neuroprotection in PD mice model by stimulating glutathione peroxidase (GPx), superoxide dismutase (SOD), Na(^+^), and K(^+^)-ATPase [141]. Quercetin suppressed cell death in PD cell model while its metabolite quercetin-3-*O*-*β*-glucuronide, due to its low absorption, did not affect cell viability [142]. Another study showed that conversion of quercetin metabolites to its aglycone in neural cells is essential for neuroprotective activity [143]. These studies had shown consistent results as compared to a contradictory report [144] which showed that quercetin had no neuroprotective role in PD cells and rat models. Other polyphenols such as baicalein [145], kaempferol [146], caffeic acid [147], and EGCG [148] have been shown to extend neuroprotection in PD studies. Similarly, polyphenolic extracts from various plants have also exhibited pharmacological role in PD studies. For instance, polyphenols-rich mulberry fruit extracts have shown antioxidant and antiapoptotic effect in SH-SY5Y cells by modulating caspase-3, B-cell lymphoma (Bcl-2), and BCL2-associated X protein (Bax) [149].

### 2.5. Huntington's Disease

CAG triplet nucleotide repeats and expanded polyglutamine region of huntingtin protein form basis of HD [30]. Polyphenols hold pharmacological relevance, as they are associated with numerous benefits including antiaging, anti-inflammatory, and anticancer effects. Resveratrol has been found to exhibit positive effects in transgenic mouse model of HD via SIRT1 activation of peroxisome proliferative activated receptor, gamma, and coactivator 1 alpha (PGC-1*α*) signaling pathway [76]. Studies have further demonstrated the Ras-extracellular signal-regulated kinase activation by resveratrol and fisetin as the basis for neuroprotection in models of HD [150]. Likewise, hesperidin and naringenin, abundant in citrus fruits, induced neuroprotection in rats possibly via nitric oxide synthase (NOS) inhibition [151]. Curcumin has been shown to control Huntington aggregates and improve various transgene-dependent parameters, thereby promising therapeutic action in HD [152]. Grape and green tea polyphenols have also exhibited potential for treating/preventing HD disease pathogenesis [153, 154]. The overall preclinical data suggests that polyphenols extend strong neuroprotection through genetic and immunological modulation, thus promising clinical prevention or delay of neurological disorders like PD and HD.

## 3. Polyphenols and Oxidative Stress

A large body of literature supports the antioxidant potential of polyphenols against oxidative stress. Resveratrol is a potent antioxidant *in vitro* [155] and *in vivo* as it attenuates oxidative stress in both animal [156] and various cell model studies [157]. Resveratrol has been shown to extend antioxidant effect by reducing the production of reactive oxygen species (ROS) and superoxide ions [158]. Similarly, quercetin has also shown protection against oxidative stress and related disorders [159]. In a variety of cell and disease models, quercetin has been shown to engage in various signaling pathways to attenuate oxidative stress and exhibit pharmacological properties [51, 114]. Polyphenol-rich green tea and its principal constituent EGCG were found to ameliorate oxidative stress in various studies [160, 161]. Other polyphenols such as puerarin [162], baicalin [163], and phloridzin [136] also attenuated oxidative stress in various disease models. Apart from *in vitro* and* in vivo* evidence, sufficient clinical evidence also suggests the antioxidant potential of polyphenols. A clinical study [164] showed that polyphenol-rich diet reduced LDL oxidation and modulated cluster of differentiation 40-ligand (CD40L) gene expression, thus controlling atherogenesis and inflammation in humans. Polyphenol-rich fruit extracts have been shown to control free radicals and ROS. Polyphenol-rich bilberry juice was found to decrease oxidative stress and inflammatory markers in humans [165]. A 13-year long clinical study indicated that higher intake of antioxidant polyphenols including flavonoids and phenolic acids helps in improving memory and has potential for inhibiting brain aging [166]. Antioxidant-rich polyphenol supplementation as beverage has also been found to decrease plasma total homocysteine, thus contributing to attenuation of AD pathology [167]. It can be concluded that polyphenols are strong antioxidants *in vitro *and* in vivo *in both animal models and humans. Clinical translation of polyphenols as antioxidant therapy is a promising approach to attenuate oxidative damage due to aging and age-related disorders. 

## 4. Polyphenols and Signal Transduction Pathways

### 4.1. Akt/P13K/mTOR Pathway

Resveratrol has exhibited neuroprotection against brain ischemia through P13K/Akt pathway by downregulating the expression of glycogen synthase kinase 3 (GSK-3*β*) and cAMP response element binding (CREB) proteins [99]. Resveratrol increased cAMP and modulated Akt pathway in cell model studies [144]. Baicalein also protects against ischemia through P13K/Akt pathway [126]. The scientific evidence suggests therapeutic intervention by polyphenols via P13K/Akt pathway (Table 1).

### 4.2. NF*κ*B Pathway

NF*κ*B is an important mediator in inflammatory process and contributes to A*β* toxicity. Flavonoids and other dietary polyphenols have shown neuroprotective effects in neuronal ischemia through NF*κ*B pathway. Flavonoids, including kaempferol, quercetin, acacetin, apigenin, and luteolin, inhibit A*β*1-40 and A*β*1-42 via NF*κ*B pathway downregulation [101]. Similarly, soybean isoflavone had reversed memory impairment in rats through decrease in NF*κ*B expression [102]. Other flavonoids such as resveratrol and baicalin inhibit A*β*-induced neural inflammation via a mechanism involving downregulation of NF*κ*B signaling pathway [103, 168]. Activation of NF*κ*B is an important event in ischemic injury and contributes to both inflammation and cell death [21]. Silymarin, a flavonolignan from milk thistle (*Silybum marianum*), has protected against cerebral ischemia by inhibiting signal transducer and activating transcription (STAT-1) pathway and NF*κ*B [105]. Tetrahydroxystilbene glucoside from *Polygonum* multiforum has protected neurons from cerebral ischemia by activating SIRT1 and inhibiting NF*κ*B signaling pathway in neurons [58]. Quercetin administration has also protected rat brain against oxidative stress and hypoxia-induced damage through NF*κ*B inhibition [106]. Similarly, catechin hydrate and fisetin have been shown to protect rat brain against ischemic injury and oxidative damage by inhibiting expression of NF*κ*B and proinflammatory cytokines such as IL-1*β* and TNF-*α* [83, 94]. It is observed that recent research advances confirm an immunomodulatory role of polyphenols as they control inflammatory response by inhibiting NF*κ*B expression. 

### 4.3. PPAR Pathway

Peroxisome proliferator activated receptor gamma (PPAR gamma) plays a role of a biomarker in cerebral ischemia as CI results in its upregulation and translocation to nucleus from the cytosol. Baicalein has reversed PPAR gamma expression and also suppresses its translocation to nucleus [104]. Resveratrol has attenuated the MMP-9 by modulating the peroxisome proliferator activated receptor (PPAR) alpha expression in hypoxia model of neurons [107]. Pterostilbene, a resveratrol derivative, has significant effect on the downregulation and normalization of PPAR-*α* expression in SAMP8 mouse model studies [108]. The scientific evidence shows that benefits associated with polyphenols in age-related and other disorders are associated with downregulation of PPAR pathway.

### 4.4. Nrf2/ARE/HO1 and HIF-1 Pathway

Nuclear factor (erythroid-derived 2-) like 2 (Nrf2) pathway is also involved in Sestrin2 (Sesn2), also known as Hi95, a p53 target gene expression which leads to encoding of antioxidant proteins [169]. Epicatechin has protected neurons against stroke and oxidative stress by upregulation of Nrf2 cascade and heme oxygenase-1 (HO1) enzyme [109]. Similarly, resveratrol has also been shown to protecte brain through increased expression of Nrf2, HO-1 expression, and downregulation of apoptotic enzymes like caspase-3 [110]. Xanthohumol, a prenylated chalcone, has demonstrated neuroprotective action by inhibiting HIF-1 pathway and it further stopped signal transduction pathway leading to apoptosis by caspases [111]. Resveratrol, apart from Nrf2 and HO-1 expression, has protected against ischemic injury in cells by downregulation of mRNA expression of hypoxia inducible factors-1*α* (HIF-1*α*) [112]. Upregulation of Nrf2 and HO-1 pathways by polyphenols in response to oxidative insult shows protective role of these compounds in brain health and oxidative damage.

## 5. Polyphenols and Immune Response

Proinflammatory cytokines and genes contribute to inflammation and neuronal death in various neurological disorders. Most of the therapeutics target cytokines and other immune responses for therapeutic intervention. Polyphenols are well known for their anti-inflammatory activities and thus control neuroinflammation and neural death. EGCG has been found to inhibit expression of monocyte chemotactic protein (MCP-1/CCL2) and IL-1*β*, thus protecting blood-brain barrier (BBB) integrity during pathological inflammation [170]. In another study, EGCG inhibited cytokine and chemokines including IL-1*β*, IL-6, and MCP-1 [116]. Resveratrol has also controlled hippocampal inflammation by reducing expression of MCP-1 mRNA levels [118]. Polyphenols, that is, catechin, caffeic acid, and transresveratrol, reduced production of inflammatory markers MCP-1, MIP-1*α*, MIP-1*β*, chemokine receptor-1 (CCR1), and CCR2 in vascular wall [119]. Polyphenol-rich olive oil controlled inflammation by inhibiting proinflammatory CD40, a costimulatory protein found on antigen presenting cells, gene expression [164]. EGCG reduced expression of inflammatory cytokines and chemokines such as chemokine (C-X-C motif) ligand (CXCL10), CCL22, CCL 17, and TGF-*β*, promising strong neuroprotection in AD and stroke [117]. Quercetin has been shown to inhibit proinflammatory cytokines like IL-1*β*, IL-6, COX-2, CD40, and TNF*α* receptor-associated factor-1 (TRAF1) [120]. A similar study [121] showed that quercetin exhibits neuroprotective effect in PC12 cells and zebrafish possibly by downregulating expression of proinflammatory genes like IL-1*β* and COX-2. Resveratrol also reduced neuroinflammation and improved memory along with IL-1*β* inhibition [115]. Resveratrol has exhibited restoration of BBB integrity and inhibited rising levels of IL-17A, T-helper 17 lymphocytes, and MMP [171]. Apple polyphenols have also reduced expression of a wide range of neuroinflammatory markers including IL-1*β*, IL-6, IL-17, 1L-22, CXCL9, CXCL10, CXCL11, and IFN-*γ*, thus providing immune-modulatory effects against inflammation [122]. Studies have also shown that blueberry and apple polyphenols can attenuate neuroinflammation and improve cognitive impairment, possibly by lowering the expression of IL-1*β* and TNF*α* in rat hippocampus [172, 173]. About 20 structurally related flavonoids have been shown to inhibit hypoxia-induced STAT3 tyrosine phosphorylation promoting cell survival [174]. Fisetin and quercetin protected neurons against LPS-induced inflammation by inhibiting TNF*α* production and JNK/Jun phosphorylation [120, 139]. Resveratrol administration during ischemic stroke inhibits neuronal damage along with reduced expression of IL-1*β* and TNF*α* [175]. Overall, polyphenols modulate immune response in neurodegenerative diseases as they induce expression of antiapoptotic factors, control neuroinflammation, and modulate cell signaling under stress (Table 2). These features of polyphenols make them strong neuroprotective candidates and support their translation from laboratory to clinical trials.

## 6. Polyphenols and Metal Ion Chelation

Iron and copper play important roles in the generation of ROS through redox cycling and subsequent neurodegeneration. Metal accumulation in brain contributes to pathology of diseases like AD, MS, PD, and HD [176, 177]. EGCG exhibited iron chelating ability in SH-SY5Y neuroblastoma cells along with the inhibition of apoptotic factors like BCL2-associated agonist of cell death (Bad), Bax, and caspase-3 [178]. EGCG has exhibited stronger chelation of iron compared to desferrioxamine and increased transferrin receptor protein along with the elevation in mRNA levels in SH-SY5Y neuroblastoma cells [179]. Electron paramagnetic resonance studies [180] demonstrated the interaction of EGCG and gallic acid as ligands to copper coordination sphere, thus demonstrating Cu modulatory potential of these polyphenols. Also, iron modulation by curcumin in rat brain homogenate has been observed, thus warranting that curcumin-based therapy in AD and PD disease models [181]. Curcumin has extended neuroprotection in a rat model of PD against 6-hydroxydopamine treatment through its iron chelating activity and reduced degeneration of neurons [182]. Similarly, curcumin's ability to reverse neurodegeneration in hemi-Parkinson's mice model has been shown [183]. Apart from iron chelation, NF*κ*B modulation by curcumin has also contributed to the reduction in 6-OHDA-induced neurodegeneration [184]. Rosmarinic acid, a phenolic acid found in Lamiaceae herbs, protected neurons against 6-OHDA treatment by lowering the expression of Bax/Bcl-2 at gene level and decreasing iron level in both MES23.5 dopaminergic cells and rat model of PD [185, 186]. It is evident that polyphenols are potent metal chelators and extend neuroprotection against iron- and copper-induced oxidative stress and neurotoxicity via metal chelation, modulation of signal transduction, oxidative stress, and inflammation.

## 7. Polyphenols and Prions

Prion proteins are involved in neurodegenerative diseases, and their conformational transition forms basis of prion diseases. The pathology of prion proteins has been inhibited by EGCG and ECG, thus exhibiting neuroprotective potential [187]. Studies have also confirmed the antiprion activity of resveratrol through autophagy activation in neuroblastoma cells [188]. Resveratrol also protected mouse neurons against PG14-PrP (mutant prion protein) expression [189]. Curcumin also downregulated the prion pathology in neuroblastoma cells [190]. Therefore, polyphenols seem to protect neurons against prion diseases by controlling prion mutation and pathology.

## 8. Polyphenol and Anti Acetylcholinesterase Activity

Pathology of neurodegenerative diseases including AD includes deficiency of neuromediator acetylcholine, thus making acetylcholinesterase (AChE) inhibitors as important clinically relevant drugs in AD and other dementia [191]. Black chokeberry extract, a rich source of polyphenols, in combination with lemon juice inhibited AChE [192]. The prenylated flavonols from paper mulberry (*Broussonetia papyrifera*) were potent inhibitors of AChE, thus exhibiting neuroprotective potential [193]. Studies have shown that a polyphenol-rich blueberry extract also inhibited AChE activity *in vitro *[194]. Polyphenols extracted from *Paulownia tomentosa *fruits exhibited inhibitory action against both AChE and butyrylcholinesterase (BChE) [195]. Quercetin was found to improve cognitive ability and exhibit neuroprotection against trimethyltin-induced neurotoxicity by inhibiting AChE [196]. A report has also shown that quercetin inhibited AChE activity and improved cognitive abilities in streptozotocin-treated mice [197]. Quercetin and macluraxanthone, from *Maclura tinctoria* and Dyer's mulberry, respectively, inhibited both AChE and BChE *in vitro* by competitive and noncompetitive inhibition, respectively [191]. Molecular docking studies [191] have indicated the hydrophobic interactions and strong hydrogen bonding of both flavonoids with enzymes as basis of their inhibitory activity. Polyphenols from *Cistus laurifolius *L. also exhibited cholinesterase inhibitory effects against AChE and BChE, supporting a neuroprotective role of polyphenols [198]. A herbal tea from *Paulownia barbatus* leaves reduced AChE activity by 40% and its principal constituent, natural polyphenol rosmarinic acid, reduces AChE activity by 25% [199]. Galangin, a flavonol isolated from rhizome of* Alpiniae officinarum, *has also exhibited strong AChE inhibition [200]. EGCG enhances huperzine A's (acetylcholinesterase inhibitor) effects against AChE as its supplementation leads to 88–91% inhibition [201]. Later reports have supported that EGCG supplementation with huperzine A improves cognitive abilities in AD [202]. Linarin, a flavonoid found in *Linaria* species, inhibited AChE activity in neuronal PC12 cells and extended potential for neuroprotection in AD and related disorders [203]. All these pieces of evidences suggest that polyphenols are potent AChE and BChE inhibitors, thus warranting neuroprotection and improved cognitive functions in AD and related dementia.

## 9. Polyphenols and Autophagy-Related Proteins 

Flavonoids such as hesperetin and hesperidin inhibited A*β*-induced glucose metabolism impairment in neurons and downregulated A*β* stimulated autophagy, resulting in improved cognitive functions [204]. Kaempferol also protected SH-SY5Y and primary neurons from rotenone toxicity through induction of autophagy [154]. Resveratrol exhibited neuroprotective effect by activating AMPK-SIRT1 autophagy pathway in PD cell model studies [205]. Brain-related autophagy studies have a wide research gap, and polyphenols have strong potential for inducing neuroprotection via autophagy and its related pathways.

## 10. Polyphenols as Neuronal Mitochondria Medicine

Polyphenols from wine are known to reduce oxidative stress and increase the expression of antioxidant enzymes like catalase, superoxide dismutase, glutathione reductase, and glutathione peroxidase [131]. Resveratrol upregulates antiapoptotic Bcl-2 protein and downregulates Bax protein expression [206]. Resveratrol also acted as mitochondrial antioxidant by elevating the levels of antioxidants trioredoxin-2 (TRX2) and X-chromosome-linked inhibitor of apoptosis protein [207]. Another study has shown that resveratrol increased expression of Bcl-2, thus preventing neuronal apoptosis [100]. Similarly, resveratrol controlled oxidative stress in PC12 cells and inhibited mitochondria-mediated apoptosis by downregulating Bax and upregulating Bcl-2 [112]. Similarly, lutein has shown protection of mice against ischemic injury by enhancing the Bcl-2 levels and downregulated Cox-2 and pancreatic ER kinase (PERK) [125]. Baicalein also regulated Bcl-2 and antagonized cytochrome c release in cytosol [126]. Similarly, ferulic acid, a phenolic acid, attenuates mitochondria apoptosis by inhibiting Bax, tBid expression, and elevating Bcl-2-like proteins [124]. Important transcription factors of ERK/Nrf2 pathway like glutamate cysteine ligase catalytic (GCLC) and glutamate-cysteine ligase, modifier subunit (GCLM), are upregulated by flavones like chrysin, apigenin, and luteolin to combat oxidative stress [129]. Glutathione peroxidase (GPx) levels were modulated by red wine polyphenols resulting in combat of oxidative stress [132]. Similarly, phenolic antioxidant 3,3′,5,5′-tetra-t-butyl-biphenyl-4,4′-diol also controlled expression of GPx and HIF-1*α* in hypoxia studies [133]. Various polyphenols like butein, phloretin, chrysin, apigenin, and luteolin activated HO-1 (HMOX1), GCLC, and GCLM through expression of ERK/Nrf2 pathway [129, 134]. Quercetin has also downregulated inflammatory cascade by lowering the expression of JNK, c-Jun, and interferon-*γ* inducible protein [135]. Similarly, p-JNK and COX-2 were downregulated by polyphenols from *Hibiscus sabdariffa* L. providing relief from oxidative stress and pathological inflammation [128]. EGCG controlled mitochondria lead inflammation by lowering transcription of JNK and activator protein-1 (AP-1) [116]. Neuroprotection through the phosphatidylinositol 3-kinase (P13K) and MAPK has also been shown by flavone glycoside [113]. Hesperidin carsonic acid, a major rosemary polyphenol, exhibited strong anti-inflammatory action in neurons under hypoxia stress by inhibiting ROS, MAPKs, caspase-3, and COX-2 [127]. Lowering of JNK serves as mitochondrial therapy not only in stroke but also in AD as JNK activation in AD brain leads to tau hyperphosphorylation and A*β* pathogenesis [208]. Curcumin and resveratrol exhibited neuroprotection through increased activity of NAD(P)H quinone oxidoreductase (NQO1) via Nrf2 pathway in astrocytes [209]. Similarly, structurally modified isomers of resveratrol also elevated the NQO1 activity, thus promising antioxidant effects through NRf2 pathway [210]. ECG modulated endophilin-B1, also known as SH3GLB1, which is required for maintaining mitochondrial morphology and plays important role in apoptosis [211, 212]. EGCG has also increased expression of mitochondrial antioxidant enzymes including superoxide dismutase (SOD) and glutathione peroxidase (GPX1) [130]. Flavonoid-enriched fraction (AF4) isolated from the peel of “Northern Spy” apples has been shown to suppress the expression of IL-1*β*, TNF-*α*, and IL-6 in a mouse model of hypoxic-ischemic (HI) brain damage [123]. Phloridzin, also an apple polyphenol, has been shown to increase expression of SOD1 and SOD2 genes, thus protecting mitochondria against oxidative stress [80]. Polyphenols are important mitochondrial therapeutics as they play a role in mitochondrial biochemistry by modulating apoptosis, antioxidant action, signal transduction, and inflammation (Table 3). 

## 11. Polyphenols and Ion Channels

The neuroprotective benefits of polyphenols are often attributed to their antioxidant activity and their ability to modulate the cell signaling pathways [105, 124, 155]. Sodium channels (Na_v_  1.5) involved in pathology of MS were found to be blocked by red grape polyphenols like quercetin, catechin, and resveratrol in rodent and cell model studies [87, 213]. G protein-coupled inwardly rectifying potassium (K_IR_3) channels, involved in neuron signaling and membrane excitability [214], are activated by naringin (flavonoid glycoside), thus exhibiting potential for improving cognition in AD [215]. EGCG's neuroprotective effect was proposed to occur through inhibition of high-voltage-activated calcium currents (*I*_HVA_) and NMDA-induced inward currents (*I*_NMDA_) along with elevation of Ca^2+^ through PLC-IP_3_ pathway [216]. Similarly, curcumin exhibited modulation of a wide range of ionic channels including Ca^2+^-release-activated Ca^2+^ channels (*I*_CRAC_), voltage-gated K^+^ channel (*I*_Kv_), intermediate-conductance Ca^2+^-activated K^+^ channel (*I*_SK4_), and the cytoplasmic Ca^2+^ concentration Δ[Ca^2+^]_C_ in Jurkat-T cells [217]. However, the current literature has a research gap of specific ion channel study (Kv3 subfamily of K^+^ channel subunits) in disease-specific conditions. Overall, the ability of polyphenols to modulate ion channels and action potential [218] complements their ability to protect neurons from disorders and thus supports the growing evidence that polyphenols may act as neuroprotectants in several neuropathological conditions.

## 12. Concluding Remarks

**Figure 2:**
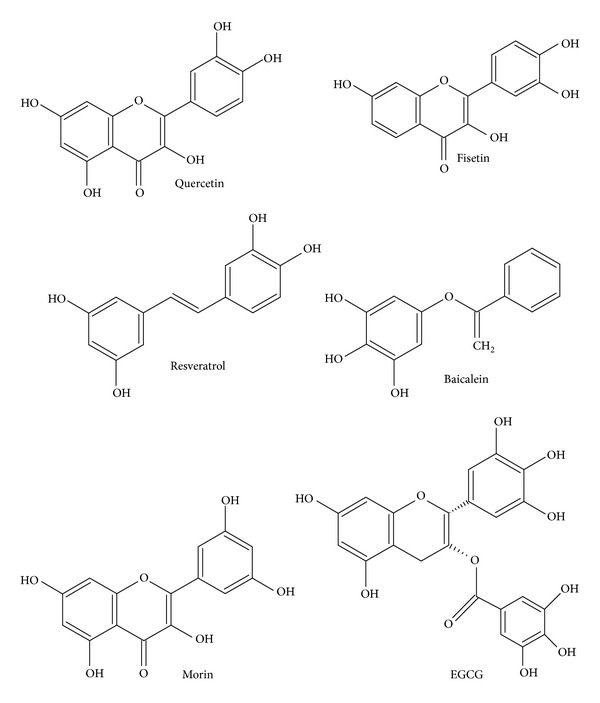
Chemical structure of polyphenols with therapeutic use in age-related neurological diseases.

In conclusion, recent scientific evidence suggests that neurodegenerative diseases are accompanied by oxidative stress, inflammation, metal accumulation, and mitochondrial dysfunctions. Various physiological mechanisms are altered by these pathological changes which contribute to etiology of neurodegenerative diseases like stroke, MS, PD, AD, and HD. The prevention and treatment of these disorders with complex mechanisms need novel therapeutic strategies targeted for multiple genes and proteins. Polyphenols are natural plant secondary metabolites which exhibit remarkable multipotent ability to control and modulate ROS, metal toxicity, inflammation, apoptosis, signal transduction, ion channels, and neurotransmitters. Polyphenolic dietary antioxidants, particularly resveratrol, EGCG, quercetin, and other fruit polyphenols, are potent neuroprotectants (Figure 2). Their direct usage and dietary supplementation could act as antioxidant and neuroprotective therapy for treatment of these diseases. Most of experimental and epidemiological studies suggest that dietary polyphenols activate antioxidant pathways such as Nrf2/HO1 and downregulate NF*κ*B, MMPs, PPAR, HIF-1, and STAT pathways. Polyphenols also modulate immune response by inhibiting proinflammatory biomarkers such as CCL17, CCL22, CCR1, CCR2, MIP1*α*, MIP 1*β*, CXCL (9, 10, 11), IFN-*γ*, TNF-*α*, and IL(1*β*, 6, 17A, 22). These salient properties of polyphenols help to reduce two hallmarks of neurodegeneration, that is, oxidative damage and inflammation. 

Polyphenols also protect mitochondria from pathological events by triggering prosurvival cell signaling. Polyphenols increase antioxidant enzymes, that is, catalase, superoxide dismutase (SOD1, SOD2), and prosurvival Bcl-2 and PERK pathways. Downregulation of Bad/Bax, c-jun, JNK, COX-2, AP-1, and caspase-3 also contributes to the survival of neurons. Polyphenols also help in improving cognitive abilities by inhibiting AChE and BChE. The inhibition of these enzymes plays an important role in clinical medicine of AD. Apart from their anti-AChE activity, polyphenols also induce metal chelation and modulate autophagy and prion proteins. These features along with reduction of A*β* toxicity, reduction of neural lesions, and activation of cell survival genes are of particular relevance to neurodegenerative diseases. The activation of novel spectrum of these molecular targets forms underlying mechanism of neuroprotection by polyphenols. The lack of toxic effect and availability from natural sources makes polyphenols as clinically relevant therapeutics in neurodegeneration. 

The future of polyphenol research needs to aim towards clinical acceptance of health claims from preclinical *in vitro *and animal model studies. Therefore, future studies focusing on human clinical trials of several potent polyphenols and their combinations should be carried out. Furthermore, polyphenols must be investigated for the risk assessment and safety evaluation to observe any undesirable effects. The success in clinical research of polyphenols will decide their pharmacological relevance for humans.

## Figures and Tables

**Table 1:** Neuroprotective signal transduction by polyphenols.

Pathway	Polyphenol	References
P13K/AkT pathway	Resveratrol	[99, 100]
	Baicalein	[97]
NF*κ*B pathway	Kaempferol, acacetin, apigenin, luteolin	[101]
	Soybean isoflavones	[102]
	Fisetin	[94]
	Resveratrol	[103]
	Baicalein	[104]
	Silymarin	[105]
	Tetrahydroxystilbene	[58]
	Quercetin	[101, 106]
	Catechin hydrate	[94]
STAT pathway	Silymarin	[105]
PPAR pathway	Baicalein	[104]
	Resveratrol	[107]
	Pterostilbene	[108]
Nrf2/HO1/ARE pathway	Epicatechin	[109]
	Resveratrol	[110]
HIF-1*α*	Xanthohumol	[111]
	Resveratrol	[112]
MAPK	Flavone glycoside	[113]
	Quercetin	[114]

**Table 2:** Modulation of cytokines and inflammatory targets by polyphenols.

Polyphenol	Target	References
EGCG	IL-1*β*	[115]
	IL-6	[116]
	MCP-1	[115]
	CXCL 10,	[116]
	CCL22, CCL 17	[117]
	TGF *β*	[117]
Resveratrol	MCP-1	[118]
Catechin	MCP-1 (*α* and *β*)	[119]
Caffeic acid	CCR1, CCR2	[119]
Quercetin	IL-1*β*, IL-6	[120]
	COX-2	[120]
	COX-20, TRAF1	[121]
Apple polyphenols	IL-1*β*, IL-6, IL-17, IL-22 CXCL-9, CXCL-10, CXCL-11,	[122, 123]

**Table 3:** Modulation of mitochondrial targets by polyphenols.

Target	Polyphenol	Effect	References
AP-1	EGCG	Downregulation	[116]
Bad/Bax	Resveratrol	Downregulation	[112]
	Ferulic acid	Downregulation	[124]
Bcl-2	Lutein	Upregulation	[125]
	Baicalein	Upregulation	[126]
Cox-2	Lutein	Downregulation	[125]
	Hesperidin	Downregulation	[127]
	*Hibiscus sabdariffa* polyphenols	Downregulation	[128]
GCLM	Chrysin, apigenin, luteolin	Upregulation	[129]
GCLC	Chrysin, apigenin, luteolin	Upregulation	[129]
GPX	EGCG	Upregulation	[130]
	Red wine polyphenols	Upregulation	[131, 132]
	3,3′,5,5′-tetra-t-butyl-biphenyl-4,4′-diol	Upregulation	[133]
HO-1	Butein, apigenin	Upregulation	[129]
	Luteolin	Upregulation	[134]
IFN-*γ*	Quercetin	Downregulation	[135]
JNK	Quercetin	Downregulation	[135]
	EGCG	Downregulation	[116]
	*Hibiscus sabdariffa* polyphenols	Downregulation	[128]
JUN	Quercetin	Downregulation	[135]
SOD	Phloridzin	Upregulation	[136]

## Abbreviations

AAP: Amyloid precursor protein
AChE: Acetylcholinesterase
AD: Alzheimer's disease
AP-1: Activator protein-1
A*β*: Amyloid beta
Bad: BCL2-associated agonist of cell death
BAX: BCL2-associated X protein
BBB: Blood-brain barrier
BChE: Butyrylcholinesterase
Bcl: B-cell lymphoma
Bcl2: B-cell lymphoma 2
cAMP: Cyclic adenosine monophosphate
CCL: Chemokine (C-C motif) ligand
CCR: Chemokine receptor
CD: Cluster of differentiation
CI: Cerebral ischemia
COX: Cyclooxygenase
CREB: cAMP response element binding protein
CXCL: Chemokine (C-X-C motif) ligand
EAE: Experimental autoimmune encephalomyelitis
ECG: Epicatechin gallate
EGCG: Epigallocatechin gallate
GCLC: Glutamate cysteine ligase catalytic
GCLM: Glutamate-cysteine ligase, modifier subunit
GPx: Glutathione peroxidase
GSK: Glycogen synthase kinase
HD: Huntington's disease
HIF: Hypoxia inducible factor
HIF-1: Hypoxia inducible factor-1
HO: Heme oxygenase
IFN*γ*: Interferon-gamma
IL: Interleukin
JNK: c-Jun N-terminal kinase
MAPK: Mitogen activated kinase-like protein
MCP-1: Monocyte chemoattractant protein-1
MIP: Macrophage inflammatory protein
MMP: Metalloproteinases
MS: Multiple sclerosis
NF*κ*B: Nuclear factor-kappa B
NOS: Nitric oxide synthase
NQO1: NAD(P)H quinone oxidoreductase
Nrf2: Nuclear factor- (erythroid-derived 2-) like 2
PC: Protein carbonyl
PD: Parkinson's disease
PERK: Pancreatic ER kinase
PGC-1*α*: Peroxisome proliferative activated receptor, coactivator 1 alpha
PI3K: Phosphatidylinositol 3-kinase
PPAR: Peroxisome proliferator activated receptor
PrP(c): Cellular prion protein
ROS: Reactive oxygen species
SIRT-1: Silent mating type information regulation 2 homolog1
SOD: Superoxide dismutase
STAT: Signal transducer and activation of transcription
TGF*β*: Transforming growth factors *β*
TLR: Toll-like receptor
TNF*α*: Tumor necrosis factor-alpha
TRAF: TNF receptor associated factor
TRX: Thioredoxin.
